# Nanoclay-Composite Hydrogels for Bone Tissue Engineering

**DOI:** 10.3390/gels10080513

**Published:** 2024-08-03

**Authors:** Hee Sook Hwang, Chung-Sung Lee

**Affiliations:** 1 Department of Pharmaceutical Engineering, Dankook University, Cheonan 31116, Republic of Korea; 2 Department of Pharmaceutical Engineering, Soonchunhyang University, Asan 31538, Republic of Korea

**Keywords:** nanoclay, nanocomposite, hydrogel, bone tissue engineering, regenerative medicine

## Abstract

Nanoclay-composite hydrogels represent a promising avenue for advancing bone tissue engineering. Traditional hydrogels face challenges in providing mechanical strength, biocompatibility, and bioactivity necessary for successful bone regeneration. The incorporation of nanoclay into hydrogel matrices offers a potential unique solution to these challenges. This review provides a comprehensive overview of the fabrication, physico-chemical/biological performance, and applications of nanoclay-composite hydrogels in bone tissue engineering. Various fabrication techniques, including in situ polymerization, physical blending, and 3D printing, are discussed. In vitro and in vivo studies evaluating biocompatibility and bioactivity have demonstrated the potential of these hydrogels for promoting cell adhesion, proliferation, and differentiation. Their applications in bone defect repair, osteochondral tissue engineering and drug delivery are also explored. Despite their potential in bone tissue engineering, nanoclay-composite hydrogels face challenges such as optimal dispersion, scalability, biocompatibility, long-term stability, regulatory approval, and integration with emerging technologies to achieve clinical application. Future research directions need to focus on refining fabrication techniques, enhancing understanding of biological interactions, and advancing towards clinical translation and commercialization. Overall, nanoclay-composite hydrogels offer exciting opportunities for improving bone regeneration strategies.

## 1. Introduction

Bone tissue engineering has emerged as a promising field aiming to develop novel strategies for regenerating damaged or diseased bone tissue [1,2,3,4]. The economic impact of long-bone fracture infection and nonunion on health-related quality of life and utility scores is significant, highlighting the extensive costs incurred by healthcare systems globally [5,6,7,8,9]. Traditional approaches to bone repair, such as autografts and allografts, have limitations including donor site morbidity, limited supply, and risk of immune rejection [10,11]. Consequently, researchers have turned to tissue engineering approaches to develop alternative solutions that could address these challenges and offer enhanced regenerative outcomes.

Hydrogels have garnered significant attention in tissue engineering due to their unique properties, including high water content, biocompatibility, and tunable mechanical properties [2,4,12,13]. These three-dimensional networks of cross-linked polymer chains closely mimic the extracellular matrix (ECM) of natural tissues, providing an ideal microenvironment for cell proliferation and tissue regeneration [14,15,16]. However, traditional hydrogels often lack the mechanical strength and bioactivity required for successful bone regeneration, prompting the exploration of novel strategies to enhance their performance.

Nanoclays such as Laponite, montmorillonite, and halloysite possess unique properties, including a high aspect ratio, large surface area, and inherent biocompatibility [17,18,19]. These nanoparticles can be dispersed within hydrogel networks to reinforce mechanical properties, improve swelling behavior, and enhance bioactivity, making them promising candidates for bone tissue engineering applications [3,16,20,21,22]. Integrating nanoclay into hydrogels offers several advantages for bone regeneration. Nanoclay reinforcement can significantly enhance the mechanical properties of hydrogels, endowing them with mechanical strength comparable to that of native bone tissue [22]. This is crucial for providing structural support and preventing the collapse or fracture of the scaffold during the healing process. Additionally, nanoclay incorporation can modulate the degradation rate of hydrogels, allowing for the controlled release of bioactive molecules to promote tissue regeneration [22,23]. Furthermore, nanoclay-composite hydrogels exhibit excellent bioactivity, facilitating cell adhesion, proliferation, and differentiation [18,24]. The presence of nanoclay within the hydrogel matrix can promote cell–material interactions through surface modification and functionalization, creating a favorable microenvironment for osteogenic cell behavior [20]. This enhanced bioactivity is essential for driving the formation of new bone tissue and accelerating the healing process for bone defects.

Using nanoclay-composite hydrogels is a promising approach for enhancing the mechanical properties, bioactivity, and regenerative capacity of hydrogel scaffolds for bone tissue engineering. By leveraging the unique properties of nanoclays, researchers can develop innovative strategies to address the limitations of traditional hydrogels and accelerate the development of advanced therapies for bone repair and regeneration.

This review sets the stage for exploring nanoclay-composite hydrogels, encompassing their fabrication, characterization, and diverse applications in bone tissue engineering. Subsequent sections of this article will delve deeper into these aspects, providing insights into advancements and challenges in this rapidly evolving field.

## 2. Hydrogels in Bone Tissue Engineering

Hydrogels have emerged as promising scaffolds for bone tissue engineering due to their unique properties that mimic the natural extracellular matrix (ECM) of bone tissue [25,26]. These three-dimensional networks of cross-linked polymer chains possess high water content, tunable mechanical properties, and biocompatibility, making them suitable for supporting cell adhesion, proliferation, and differentiation [2,25]. In the context of bone tissue engineering, hydrogels serve as platforms for delivering therapeutic agents, supporting cell growth, and promoting tissue regeneration.

**Figure 1:**
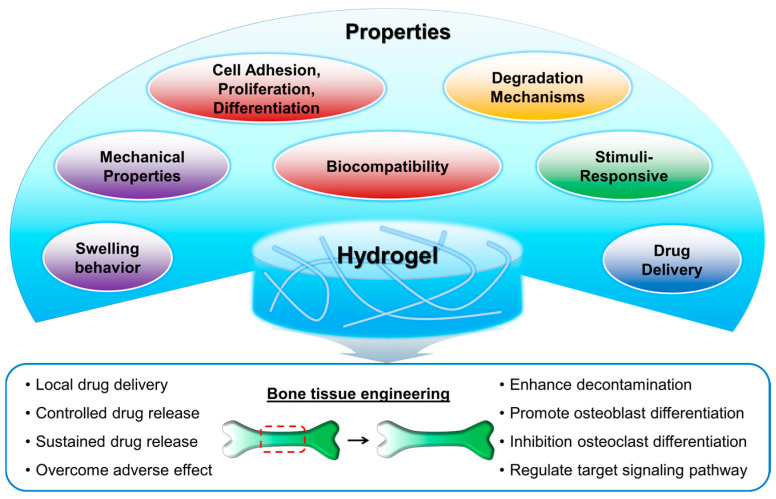
Properties of hydrogels for bone tissue engineering.

Hydrogels exhibit a range of properties that make them attractive for various applications, including tissue engineering and drug delivery. One of the most distinctive properties of hydrogels is their ability to swell in aqueous environments [27,28]. This swelling behavior arises from the hydrophilic nature of polymer chains known to have a high affinity for water molecules. As water is absorbed into the hydrogel matrix, polymer chains will expand, leading to an increase in volume. The extent of swelling can be controlled by adjusting parameters such as polymer composition, cross-linking density, and environmental conditions including pH and temperature [29,30,31]. In addition, hydrogels possess tunable mechanical properties that can be tailored to match the stiffness and elasticity of various biological tissues [32,33,34]. The mechanical strength of hydrogels is influenced by factors including polymer chain length, cross-linking density, and the presence of reinforcing agents such as nanoparticles and fibers [35,36,37,38]. By modulating these parameters, hydrogels can be engineered to provide mechanical support for tissue regeneration while maintaining flexibility and compliance with surrounding tissues. Furthermore, hydrogels are generally biocompatible materials that support cell adhesion, proliferation, and differentiation [39,40]. The hydrophilic nature of hydrogel surfaces can minimize non-specific protein adsorption and reduce the risk of immune rejection [41]. The use of porous polyethylene glycol diacrylate (PEGDA) hydrogels, known for their tunable porosity and mechanical strength, can also be highlighted as promising options for supporting bone tissue regeneration [42]. The porous structure of hydrogels allows for the diffusion of nutrients and oxygen to encapsulated cells, promoting cell viability and function [20]. Additionally, hydrogels can undergo degradation over time through various mechanisms, including enzymatic cleavage, hydrolysis, and erosion [43,44,45,46]. The degradation kinetics of hydrogels can be tailored by selecting appropriate polymer chemistries and cross-linking strategies. Controlled degradation is essential for tissue engineering applications as it allows for gradual remodeling of a scaffold and integration with surrounding tissues [16,47,48]. Some hydrogels exhibit responsiveness to external stimuli such as pH, temperature, and light [49,50,51,52,53,54]. These stimuli-responsive hydrogels can undergo reversible changes in swelling, stiffness, and/or porosity in response to changes in their environment. This reversible property of hydrogels has led to the development of smart hydrogel systems for controlled drug delivery, biosensing, and tissue engineering applications [50,51]. In addition, it is important to combine and formulate a variety of potential biomaterials, such as the combination of gelatin and hydrocolloids, which provide enhanced mechanical properties and biocompatibility for hydrogel formulations and materials [55]. Hydrogels offer a versatile platform for a wide range of biomedical applications due to their unique properties, including swelling behavior, mechanical tunability, biocompatibility, degradation kinetics, and responsiveness to external stimuli (Figure 1). Understanding and harnessing these properties are essential for designing and developing hydrogel-based biomaterials with tailored functionalities for specific biomedical applications.

## 3. Nanoclay Reinforcement of Hydrogels

Nanoclay reinforcement of hydrogels involves the incorporation of nanoclay particles into hydrogel matrices to enhance their mechanical properties and performances. Nanoclays are naturally occurring or synthetic silicate nanoparticles, and offer unique advantages such as high aspect ratios, large surface areas, and excellent mechanical properties [56,57,58,59]. When dispersed within hydrogel networks, nanoclays can reinforce the structure, improve mechanical strength, and provide additional functionalities to the resulting nanocomposite materials [59,60]. This reinforcement mechanism occurs through interactions between the nanoclay particles and the polymer chains of the hydrogel matrix, leading to enhanced load-bearing capacity and resistance to deformation. The process of incorporating nanoclays into hydrogels can vary depending on factors such as the type of nanoclay, hydrogel composition, and desired properties of the resulting nanocomposite material [60]. Common methods include physical mixing, solution casting, and in situ polymerization. During the fabrication process, it is crucial to achieve uniform dispersion of nanoclays within the hydrogel matrix to maximize their reinforcing effects and ensure consistent mechanical performance throughout the material. Nanoclay nanoparticles used for reinforcing hydrogels typically have diameters ranging from 20 to 200 nm. The specific diameter can vary depending on the type of nanoclay and the preparation methods used. These particles offer high aspect ratios and large surface areas, which are critical for enhancing the mechanical properties of hydrogels through better interaction with the polymer chains within the hydrogel matrix [20,22]. This interaction results in improved load-bearing capacity and resistance to deformation [61].

Hydrogel scaffolds for use in tissue engineering must be structurally stable to withstand in vivo mechanical stress while supporting ingrown tissues [62]. Enhanced mechanical toughness by incorporating nanoparticles between polymer chains, which increases the entanglement of the polymer network, is an important property of biomaterials used in regenerative medicine [63]. Nanoclays can significantly improve various mechanical properties of hydrogels, including tensile strength, elastic modulus, toughness, and fracture resistance [64,65,66,67]. These enhancements stem from the reinforcement of the hydrogel network by nanoclay particles, which can act as physical cross-linkers and hinder the propagation of cracks and defects within the material. Zhai et al. reported nanoclay-composite hydrogels with poly(4-acryloylmorpholine) and Laponite nanoclay [64]. The incorporation of nanoclays into hydrogels enhanced mechanical properties such as tensile strength, Young’s modulus, compressive modulus, and elongation rate. These properties were increased when the nanoclay content was increased from 1% to 7% (≈50-fold of tensile strength; ≈9-fold of elongation; ≈30-fold of Young’s modulus; 5-fold of compressive modulus). Furthermore, the mechanical strength of the nanoclay composite hydrogel was found to be influenced not only by the nanoclay but also by the polymer concentration. It was confirmed that a higher polymer concentration results in greater mechanical strength. However, elongation at break was decreased when nanoclay content was higher (i.e., 7% nanoclay) than the 5% group, suggesting that higher nanoclay concentration could affect the toughness of the hydrogel and make the hydrogel slightly brittle. Additionally, nanoclays can impart thermal stability and stimuli-responsive behavior to hydrogels, further expanding their potential applications in areas such as drug delivery and tissue engineering [28,68,69,70].

Several polymers can physically interact with nanoclays, significantly affecting the swelling ability of hydrogels. The ability to retain specific molecules combined with stimulus-responsive swelling, water expulsion, absorption, and content release supports the utility of hydrogels as promising platforms for therapeutic drug delivery [63]. Most previous studies have reported that the swelling ability of hydrogels decreases with the incorporation of nanoclay [20]. In a recent study, Panahi et al. [71] evaluated the effect of MMT incorporation on water absorption capacity of chitosan/polyvinylpyrrolidone-based hydrogels. Increasing the MMT content from 3 wt.% to 13 wt.% improved the swelling capacity of the hydrogel. This can be considered a unique and very interesting finding, different from results of most previous nanoclay-composite hydrogel studies. However, a significant decrease in swelling ability occurred when MMT exceeded 13 wt.%. When MMT content was increased beyond 18 wt.%, the physical cross-linking density was excessively enhanced. However, the water absorption capacity decreased. Datta Chaudhuri et al. [72] observed an increase in swelling similar to the profile of starch-grafted polyacrylic acid-based hydrogels with addition of bentonite nanoclay.

For injectable hydrogels, it is crucial to have a precursor solution that is easily injectable and capable of gelation at the site of application to form a mechanically stable hydrogel. This property makes injectable hydrogels particularly valuable for minimally invasive medical procedures. Rheological characterization can be used to evaluate properties such as injectability, flow behavior, and gelation temperature, along with other characteristics of nanocomposite hydrogels. Huang et al. [73] developed PEG hydrogels strengthened with functionalized layered double hydroxide (LDH) nanoclays. They observed that as the LDH content increased, the storage modulus was significantly increased. Similar results have also been observed by Lee et al. [22] for Laponite-composite chitosan-based hydrogels. This clearly demonstrates that the addition of nanoclay can influence the viscoelastic properties and overall structural integrity of hydrogels. Su et al. [74] improved the gelation of injectable silk fibroin hydrogels using Laponite. Typically, the gelation of silk fibroin aqueous solution takes several weeks at 4 °C, approximately 10 days at 37 °C, and approximately 40 min at 95 °C. The addition of Laponite significantly reduced the gelation time of the silk fibroin solution at 37 °C from 10 days to under 3 h. Moreover, Zhang et al. [21] developed a guanidine-modified chitosan–Laponite composite hydrogel, which can form a gel via electrostatic interaction between cationic chitosan polymers and anionic Laponite nanoclays. Increasing Laponite content to more than 4% resulted in fast gelation within 10 s.

For tissue engineering applications, hydrogel scaffolds need to be structurally robust to withstand in vivo stresses while facilitating tissue growth. The addition of nanoparticles within the polymer matrix can enhance structural and mechanical toughness by increasing polymer network entanglements. This improvement in mechanical properties is crucial for biomaterials utilized in regenerative medicine. Cross et al. [75] developed a gradient scaffold made of GelMA and methacrylated carrageenan reinforced with Laponite nanoclays, leading to a notable enhancement in hydrogel strength. Specifically, adding 1 wt.% Laponite increased the compressive strength of hydrogels. This improvement is attributed to the diffusion of polymeric chains into the basal space of silicate layers, resulting in strong interfacial interactions [20]. Consequently, nanoclay-composite hydrogels are believed to be able to withstand greater external loads than only polymer hydrogels.

The degradability of biomaterial systems is a fundamental characteristic with significant effects on biomedical applications. In tissue engineering, hydrogel scaffolds must provide and maintain the needed structural support for the duration required for new tissue regeneration. This characteristic ensures that the hydrogel scaffold degrades in sync with tissue growth, avoiding premature loss of support and minimizing surgical interventions for scaffold removal. Similarly, in drug delivery applications, controlled degradability is crucial to preventing premature burst release of therapeutic agents, ensuring a sustained and effective delivery over the desired period [69,76,77,78]. Kerativitayanan et al. developed poly(glycerol sebacate) hydrogels enhanced with Laponite nanoclays for tissue engineering [76]. These nanocomposite hydrogels exhibited a notably slow degradation rate. The researchers found that incorporating nanoclays led to a higher degree of cross-linking, slower weight loss, and better physiological stability. Their findings indicate a strong link between the inclusion of nanoclay particles and enhanced degradation properties. Following their research, Lee et al. [22] similarly developed polyphenol-modified chitosan hydrogels with Laponite nanoclay-composites for bone tissue engineering and achieved significantly reduced degradation. Mauro et al. [79] also reported that polyamidoamine–montmorillonite hydrogel composites designed for bone tissue applications exhibit extended degradation time when clay nanoparticles are incorporated.

The microstructure of a hydrogel’s matrix plays a crucial role in mass transport mechanisms. This microstructure facilitates the delivery of biological agents and enables dynamic interactions and exchanges with native cellular environments, which are essential for tissue regeneration within the hydrogel scaffold. The efficiency of mass transport is influenced by factors such as pore size, shape, and free volume, all of which are directly dependent on the hydrogel’s architecture. These characteristics determine how well the scaffold can support cellular activities and integrate with host tissues, making them vital for effective tissue engineering applications [22,64,80].

Cui et al. [20] observed the microstructure of nanoclay-composite chitosan hydrogels. They confirmed that microporous and interconnected networks were formed in hydrogels containing more than 1.5% montmorillonite. In the group containing 3.0% montmorillonite, the pore size increased further. The unique structure of nanoclay contributed to the formation of microstructure by forming pores and inter-particle spaces through interaction with polymer chains. This porous microstructure plays a very important role in tissue formation processes, including matrix deposition, vascularization, cell invasion, and cell proliferation. In another study, Huang et al. [81] discussed various benefits of adding nanoclay, specifically halloysite nanoclay, to sodium alginate hydrogels, focusing on their morphology and potential applications. Nanocomposite hydrogels developed by them exhibited pore sizes ranging from 100 to 250 μm, with a slight reduction in size compared to sodium alginate hydrogels without nanoclay addition. A pore size of around 200 μm was found to be advantageous for uniform cell seeding and penetration, supporting cell growth, proliferation, and interactions with materials, while also providing enough space for extracellular matrix secretion. Additionally, the roughness of the pore surface could be adjusted by varying the concentration of nanoclays, enhancing cell adhesion. Additionally, they found that incorporating nanoclays did not alter the crystalline structure of the nanoclay, as evidenced by the diffraction spectrum which showed no new peaks or changes in peak locations.

For designing a drug delivery system, various parameters and properties, such as processing conditions, surface properties, morphology, and porosity, can affect not only drug encapsulation efficiency, but also the drug release mechanism. In nanocomposite hydrogel systems incorporating nanoclays, release mechanisms can be influenced by polymer swelling or degradation, the creation of diffusion barriers, and interactions between the polymer, nanoclay, and water [57,58,59,63,69]. These interactions can significantly dictate the overall drug release profile. Li et al. [82] demonstrated that PEGylated biomacromolecules (IGF1 mimetic proteins) show significantly higher binding capacity than typical hydrogel drug loadings because clay minerals can maintain their high surface area and charge density even after forming nanocomposite hydrogels. This binding capacity was due to electrostatic interactions between positively charged IGF1 and negatively charged nanoclay surfaces. In systems with 6% and 8% nanoclay, a nearly zero-order release profile with minimal initial burst was observed. In contrast, alginate hydrogels without nanoclay showed a substantial initial burst release of >60% and a release duration of fewer than three days. These results suggested that clay nanoparticles played a crucial role in regulating the protein release rate by interacting with protein molecules that are generally smaller than the mesh size of the alginate hydrogel network. In another study, the same conclusions were observed by Hua et al. [83], who developed a series of chitosan hydrogels containing ofloxacin and montmorillonite (nanoclay) to evaluate the impact of nanoclays on the encapsulation and delivery of therapeutic agents. They found that the incorporation of nanoclay enhanced the encapsulation efficiency of ofloxacin compared to hydrogels without nanoclay. This improvement is likely due to the large surface area of nanoclay, which can adsorb ofloxacin on both its surface and within its interlayer spaces. Drug release from these hydrogels was influenced by swelling and degradation. Additionally, the presence of nanoclay facilitated a slower and more continuous release of ofloxacin.

The interaction of hydrogels with cells, such as mesenchymal stem cells, is a crucial factor influencing cell survival, proliferation, and differentiation. For example, hydrogels with cell adhesion sites can significantly increase cell viability [16,84,85]. Introducing nanoclays into these hydrogels can result in stem cells with round and spherical morphology due to inadequate interaction with the polymer matrix. However, proper concentrations of nanoclays can lead to better cytoskeletal organization and cell spreading. This indicates that nanoclay concentration has a clear relationship with cell adhesion, cell spreading, and cytoskeletal structure. When polymer chains in nanocomposite hydrogels hydrate, nanoclay surfaces are exposed, which can serve as cell adhesion sites [20,86]. Additionally, adding nanoclay to a polymer network to control cell adhesion is a straightforward, cost-effective, and reproducible method. Dong et al. [87] created Laponite-reinforced gelatin methacrylate (GelMA) cross-linked hydrogels for 3D printing and bone regeneration applications. Their research showed that cells could adhere to and proliferate on the surface of these nanoclay-composite hydrogels. Osteogenic differentiation of bone marrow stromal cells (BMSCs) was also observed in vitro. This was attributed to enhanced protein adsorption facilitated by electrostatic interactions with Laponite, which improved cell adhesion and proliferation. The incorporation of nanoclay significantly boosted alkaline phosphatase activity and Alizarin Red S staining. Their study revealed positive correlations of Laponite concentration with cell differentiation and calcium deposition. These findings suggest that adding high concentrations of Laponite can induce cell adhesion and differentiation without the need for growth factors.

**Figure 2:**
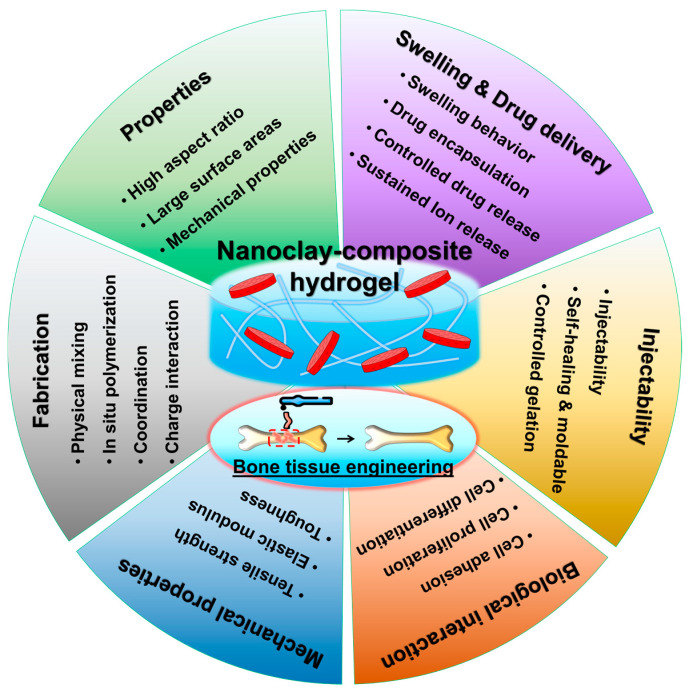
Considerations and benefits of nanoclay reinforcement in hydrogels for bone tissue engineering.

The choice of nanoclay type, concentration, and dispersion method can influence the final properties and performances of nanoclay-reinforced hydrogels (Figure 2) [28]. For example, layered silicate nanoclays such as montmorillonite and Laponite are commonly used due to their high aspect ratios and compatibility with hydrogel matrices. The concentration of nanoclays can be tailored to achieve the desired balance between mechanical reinforcement and other functional characteristics. Moreover, surface modification techniques can be employed to enhance the compatibility and interaction between nanoclays and hydrogel polymers, further optimizing the properties of nanocomposite materials.

## 4. Recent Innovations and Applications in Bone Tissue Engineering

Applications in bone tissue engineering involve the utilization of hydrogels as scaffolds or carriers for promoting bone regeneration and repair [2,4,16,25,88]. Hydrogels offer a versatile platform for delivering bioactive molecules, supporting cellular growth, and mimicking the extracellular matrix environment, making them well-suited for addressing various challenges in bone tissue engineering [2,4,25,89]. In this chapter, we discuss recent studies on nanoclay-composite hydrogels categorized by types of nanoclay, focusing on their applications and implications in bone tissue engineering.

Laponite nanoclay is a synthetic disc-shaped phyllosilicate clay, part of the smectite group [90]. Its production from inexpensive raw materials makes it suitable for a wide range of applications. Laponite nanoclay discs have negatively charged faces and positively charged rims [91]. When hydrated, these nanoclays can form a thixotropic gel network through Van der Waals interactions [92]. This network forms more rapidly and develops distinct structures when the electrolyte concentration approaches physiological levels [93]. Numerous studies have explored the bioactive properties of Laponite nanoclay, particularly its capability to promote osteogenic differentiation of preosteoblasts, human mesenchymal stem cells (hMSCs), and human adipose-derived stem cells (hASCs), without needing growth factors [21,94,95] (Table 1). Initially, it was confirmed that nanoclay did not exhibit cytotoxicity or compromise cell membrane integrity. Subsequent differentiation assays revealed that Laponite nanoclay exposure significantly enhanced alkaline phosphatase (ALP) activity in stem cells. Furthermore, expression levels of osteogenic markers such as RUNX2, osteocalcin (OCN), and osteopontin (OPN) were substantially increased after Laponite nanoclay exposure compared to those in control groups. Particularly, self-healing chitosan-based hydrogels with Laponite nanoclays by a supramolecular electrostatic interaction can improve cell adhesion and support osteogenic differentiation of mesenchymal stem cells by activating the Wnt/β-catenin signaling pathway [21,88]. Additionally, they can serve as adaptable carriers for demineralized bone matrix (DBM). The incorporation of DBM can maintain the self-healing and injectable properties of hydrogels while boosting their osteogenic potential. This suggests that advanced allograft bone formulations with such carriers can improve handling and bone regeneration. While the precise mechanisms remain unclear, these studies suggest that the dissociation of nanoclay into individual ions (Li^+^, Mg^2+^, and Si(OH)_4_) may play a key role [59]. These ions are known to regulate various cellular processes, including the promotion of osteogenesis.

Significantly, the bioactive properties of nanoclay remain intact when integrated into synthetic polymer matrices. In addition to improving the mechanical strength of these engineered scaffolds, nanoclay can also enhance cell adhesion and promote cell differentiation. This combination of properties makes nanoclay a valuable component in the development of advanced biomaterials.

Lee et al. [22] reported a multifunctional Laponite nanoclay-composite hydrogel for bone regeneration. The integration of phytochemical-derived catechol-modified chitosan with Laponite nanoclay created biocompatible and osteoconductive mimics of the extracellular matrix. This resulting hydrogel showed antibacterial, antioxidant, and osteogenic activities resulting from the antibacterial and antioxidant properties of phytochemical moieties that could act as intermolecular networking agents to facilitate gelation. This composite material is injectable with self-healing properties. Furthermore, this nanoclay-composite chitosan hydrogel can induce osteoinductive effects by regulating the Wnt/b-catenin pathway through Laponite nanoclays. Importantly, this regulation, combined with the osteoinductive signals of the Hedgehog signaling pathway by a small molecular agent, the smoothened agonist (SAG), can promote in vivo bone regeneration in a non-healing cranial defects mouse model.

A hybrid scaffold with Bioglass and hydrogel was developed by Zheng et al. [96]. The scaffold functionalized with nanoclay (Laponite) significantly enhanced the scaffold’s mechanical properties and osteogenic differentiation of human adipose mesenchymal stem cells. The immobilization of gelatin hydrogel containing deferoxamine (DFO), a hypoxia-mimicking agent, on the BG-XLS scaffold, enabled a sustained release of DFO while preventing its degradation. The delivery of DFO facilitated bone regeneration by activating the hypoxia-inducible factor-1 alpha (HIF-1α) pathway, which promoted angiogenesis. Moreover, these scaffolds significantly improved bone healing in a mouse model with a critical-sized cranial bone defect in vivo.

**Figure 3:**
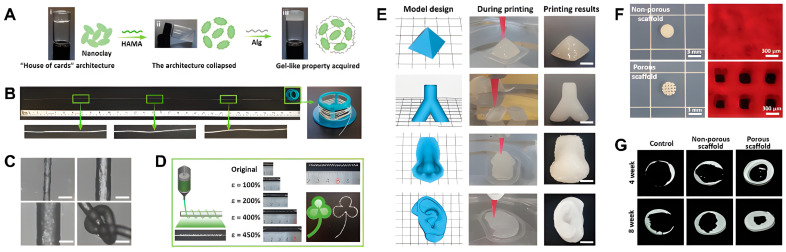
Nanoclay-composite hydrogel for 3D printing biomaterial ink. (**A**) Synthesis process and representative images of a nanoclay sol-like mixture, a nanoclay-methacrylated hyaluronic acid mixture, and a nanoclay-methacrylated hyaluronic acid-alginate gel-like mixture. (**B**) Representative images of a straight filament and straight filament rotating around the collecting rod. (**C**) Optical images of straight filaments with diameters of 100, 200, and 300 µm, respectively. (**D**) Filaments can be formed into spiral shapes to endure extensive stretching, subsequently used to create a handmade clover. (**E**) 3D printing of complex architectures based on hydrogels. (**F**) Macroscopic and fluorescence images of the non-porous scaffold and 3D printed porous scaffold based on hydrogels. (**G**) In vivo micro-CT reconstructed images of calvarial defects at 4 weeks and 8 weeks after implantation. Reproduced with permission from Guo et al. [97].

Extrusion-based 3D printing stands out among 3D printing technologies due to its ease of use and high efficiency, particularly in tissue engineering applications. In this method, a suitable biomaterial ink is essential. It must reliably extrude and retain the integrity of the printed structures throughout the printing process. Guo et al. [97] developed a novel biomaterial ink that is self-supporting, self-recovering, and 3D printable, incorporating Laponite nanoclay into a double-network hydrogel (Figure 3). This ink can be used to fabricate mechanically strong 1D filaments and 3D constructs. In these hydrogels, the commonly utilized “house-of-cards” structures conventionally formed by nanoclay or nanoclay-composite hydrogels are broken down (Figure 3A). Nonetheless, nanoclay can function as physical cross-linkers, interacting with polymer chains of methacrylated hyaluronic acid (HAMA) and alginate (Alg), which impart excellent structural formability to hydrogel precursors. This allows for the successful fabrication of various forms such as straight filaments, spring-like loops, and intricate 3D constructs with high shape fidelity and mechanical strength (Figure 3B–E). Additionally, this hydrogel system can be easily converted into a novel type of magnetic responsive hydrogel suitable for 3D printing. These resulting hydrogels can also promote the growth of bone marrow mesenchymal stem cells and show potential for repairing calvarial defects in vivo (Figure 3F,G).

**Figure 4:**
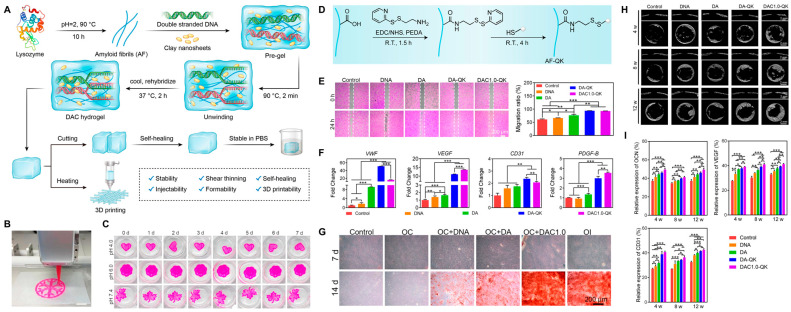
Dual nanoengineered DNA hydrogel (DAC) hydrogel for vascularized bone regeneration. (**A**) Schematic illustration of preparation of DAC hydrogel. (**B**) Schematics of the extrusion 3D printing process, scale bar: 5 mm. (**C**) Stability of 3D-printed DAC hydrogels at different pH values. (**D**) Reaction scheme of QK peptide-conjugated amyloid fibrils (AF-QK). (**E**) Stimulation effects of various hydrogels on HUVEC migration (**Left**). Quantitative migration ratio after HUVECs being cultured for 24 h (**Right**). (**F**) qRT–PCR analysis of angiogenesis-related gene (VEGF, PDGF-B, CD31, and VWF) expression in HUVECs cultured for 3 days. (**G**) Mineralized matrix determined by Alizarin red staining for 7 and 14 days. (**H**) Representative micro-CT images with cross-section and longitudinal section of skull defects implanted with hydrogels. (**I**) Quantitative analysis of OCN, CD31, and VEGF expression at 4, 8, and 12 weeks. *, *p* < 0.05; **, *p* < 0.01; ***, *p* < 0.001. AF = amyloid fibrils, DA = DNA/AF hydrogel, DAC1.0 = DNA/AF/nanoclay hydrogel. Reproduced with permission from Yang et al. [98].

Recently, Yang et al. [98] introduced a dual nanoengineered DNA dynamic hydrogel created through the supramolecular co-assembly of amyloid fibrils and Laponite nanoclays with DNA strands (Figure 4). This hydrogel is designed by mimicking an extracellular matrix-like fibrillar network that can be easily formed without complex molecular synthesis. The combination of amyloid fibrils and nanoclays can enhance the hydrogel’s mechanical strength and stability, providing features such as shear-thinning, injectability, self-healing, self-supporting, and 3D printability. Additionally, the QK peptide, an alternative VEGF mimetic peptide, is chemically grafted onto amyloid fibrils. Its sustained release from the hydrogel matrix can promote tube formation and migration of human umbilical vein endothelial cells. The hydrogel can also facilitate osteogenic differentiation of bone marrow mesenchymal stem cells through sustained release of Si^4+^ and Mg^2+^ from nanoclays. Furthermore, incorporating nanoclays along with AF-QK delivery within the hydrogel synergistically promoted both osteogenesis and angiogenesis, resulting from significantly increased expression of osteogenic marker OCN, vascularization marker CD31, and VEGF in the bone defect area. The enhanced vascularized bone regeneration by this dynamic hydrogel is demonstrated in a rat cranial bone defect model.

Integrating Laponite into hydrogels provides significant benefits, while there are no clinical studies on nanoclay-composite hydrogels according to a search result on https://clinicaltrials.gov/ (accessed on 30 July 2024). Very recently, Laponite-composite hydrogels received the 510 (k) approval from the U.S. Food and Drug Administration (FDA) [99,100]. This approval underscores the biocompatibility and functionality of this approach and its promising clinical applications. While nanoclays have been approved as additives in various pharmaceuticals by regulatory agencies, nanoclay-based composite hydrogels have not yet received such approval. Consequently, it is crucial to conduct a comprehensive review of the biocompatibility and functionality studies associated with nanoclays to advance their safe and effective use in pharmaceutical applications.

**Table 1:** Laponite nanoclay-composite hydrogels for bone tissue engineering.

Type of Nanoclay	Type of Hydrogel	Bioactive Agent	Features	Ref.
Laponite	Guanidine modified glycol chitosan	Demineralized bone matrix	Self-healing (injectable) and pro-osteogenic property, malleable carrier for the demineralized bone matrix (DBM), easy to handling, enhanced cell adhesion, activation of Wnt/β-catenin signaling pathway, and robust in vivo bone regeneration in a mouse calvarial defect model	[21,88]
Laponite	Catechol-modified glycol chitosan	Smoothened agonist (SAG)	Phytochemical-conjugated chitosan and nanoclay composite hydrogel via coordination and oxidation, porous structure by addition of nanoclays, self-healing and moldable properties, antibacterial activities against Gram-negative and Gram-positive bacteria, antioxidant activities against hydrogen peroxide and 2,2-diphenyl-1-picrylhydrazyl (DPPH), osteogenic agent delivery of nanoclays, and In vitro osteogenic activities and in vivo bone healing by Wnt/β-catenin and Hedgehog signaling pathway	[22]
Laponite	Polyethylene-glycol diacrylates	None	Approximately ~0.05 MPa of Young’s modulus, sustained release of magnesium ions and silicon ions from the nanoclay-composite hydrogel, and on vivo new bone formation in a rat tibia defect model	[101]
Laponite	Gelatin methacryloyl, 45S5 Bioglass, Polycaprolactone	Deferoxamine (DFO, an iron chelator and hypoxia mimicking agent)	Sustained releasing DFO, which in turn stimulates VEGF expression in stem cells and promotes osteogenesis of stem cells in vitro and cranial bone formation in vivo with the combination of nanoclay and Bioglass	[96]
Laponite	Gelatin methacryloyl	None	Nanocomposite hydrogel consisting of 15% gelatin methacryloyl and 8% Laponite improved the hydrogel’s degradation stability and mechanical properties by composite of nanoclay, excellent 3D-printability at room temperature due to its shear-thinning behavior, and in vitro good biocompatibility and osteogenic activities	[87]
Laponite	*N*-acryloyl glycinamide	None	Nanocomposite hydrogel as a hybrid bioink for 3D printing using *N*-acryloyl glycinamide and nanoclay enhanced mechanical properties and swelling stability of the hydrogels by combination of hydrogen bonding and physical cross-linking with nanoclay, sustained release of Mg^2+^ and Si^4+^ ions from hydrogels that can promote osteogenic differentiation of primary rat osteoblast cells and facilitate new bone regeneration in rat tibia defects	[102]
Laponite	4-acryloylmorpholine	None	Superior mechanical properties with maximum tensile strength of 0.513 MPa and Young’s modulus of 0.138 MPa from the hydrogen bonding between the poly(4-acryloylmorpholine) chains and the physical cross-linking provided by the nanoclay, excellent biocompatibility, and controlled release of Mg^2+^ and Si^4+^ ions from the hydrogel that enhances its ability to support the osteogenic differentiation of primary rat osteoblasts and in vivo new bone formation of rat tibia defects	[64]
Laponite	Methacrylated hyaluronic acid, alginate,	None	High 3D shape accuracy and mechanical strength, magnetically responsive hydrogel for 3D printing applications, biocompatible for the growth of stem cells, and in vivo calvarial defect repair	[97]
Laponite	Gelatin methacryloyl	Extracellular vesicles (EV)-derived from TSA *-treated osteoblasts	Promotes osteoinductive potency with engineered osteoblast-derived EVs, improves the retention and control delivery of bioactive factors, Laponite nanoclay dose-dependent increase in hydrogel compressive modulus and shear-thinning properties, and enhanced proliferation (1.09-fold), migration (1.83-fold), histone acetylation (1.32-fold), mineralization (1.87-fold), and extracellular matrix collagen production (≥1.3-fold)	[103]
Laponite	Sodium alginate, gelatin	None	Enhances 3D printability and mechanical strength of the hydrogel bioink after incorporating nanoclays, promotes osteogenesis in encapsulated rat bone marrow stromal cells without additional osteoinductive factors, excellent bone healing properties without any adverse effects in vivo	[104]
Laponite	Silk fibroin	None	Improve hydrogel’s mechanical properties and hydrophilicity with the addition of nanoclays, facilitate osteogenic and chondrogenic differentiation of stem cells, and support regeneration of both cartilage and subchondral bone in vivo	[105]
Laponite	Gelatin, calcium phosphate bone cement (CPC)	None	Designed for bone regeneration-adapted degradability to match the bone regeneration rate, good osteoinduction, osteoconduction, and angiogenesis, capable of complete transformation from implant to new bone, induction of ectopic bone regeneration, and promote ligament graft osseointegration in vivo	[106]
Laponite	Double stranded DNA, QK peptide-conjugated amyloid fibrils	QK peptide	Nanocomposite hydrogel can be created without complex molecular synthesis, with strength and stability boosted by amyloid fibrils and nanoclays, showing shear-thinning, injectability, self-healing, self-supporting, 3D printable properties. It can be chemically grafted onto hydrogels for controlled release of QK peptide, stimulating endothelial cell functions, promoting stem cell differentiation through ion release of nanoclays, and improving vascularized bone regeneration in a rat cranial bone defect model	[98]
Laponite	Alginate, hydroxyapatite	None	Tuned hydrogel’s physical properties by Laponite concentration control, excellent osteoinductive ability and bone-enhancing properties, and possible to inject into the sub-periosteum for bone augmentation	[107]
Laponite	Gelatin methacryloyl	SDF-1α	Incorporation of nanoclay and SDF-1α can boost osteogenic capacity and MSC homing with easy injection capability, sustained release of SDF-1α, and good ability to stimulate bone formation both in vitro and in vivo	[108]

* Trichostatin A (histone deacetylase inhibitor).

Montmorillonite (MMT) is a layered silicate mineral with a chemical formula of [(Na,Ca)_0.33_(Al,Mg)_2_Si_4_O_10_(OH)_2_·*n*H_2_O] [109]. It belongs to the smectite group. It has a high specific surface area and a high aspect ratio. The structural unit of MMT consists of an alumina octahedral sheet sandwiched between two silicon tetrahedral layers, resulting in a negative surface charge due to the dominance of oxide anions. Various studies have shown that incorporating MMT into biomaterials such as gelatin, collagen, silk, and chitosan can enhance cell-scaffold interactions, promote cell proliferation, and improve cell differentiation [20]. Particles of MMT are plate-shaped, typically around 1 nm thick and 0.2 to 2 μm in diameter. MMT is known for its biocompatibility, availability, and versatility, making it a popular subject of research, especially for drug and gene delivery applications [110]. The Food and Drug Administration (FDA) approved MMT as a biocompatible clay material widely used in pharmaceutical and industrial fields. It can enhance drug release by strongly adsorbing drug molecules. It can also improve the bioavailability and dissolution rate of hydrophobic drugs. Numerous studies have explored physico-chemical and bioactive properties of MMT nanoclay-composite hydrogels, particularly its capability to promote mechanical and osteogenic properties (Table 2).

Methacrylated glycol chitosan (MeGC)–montmorillonite (MMT) hydrogels have been developed by introducing two-dimensional nanoclay particles through intercalation chemistry [20]. Cui et al. [15] demonstrated that the presence of MMT in MeGC hydrogels enhanced the thermal stability of hydrogels and changed the microstructure of MeGC hydrogels by changing the formation of a microporous structure and interconnected network in chitosan-based in situ hydrogel. MMT with 1.5% *w*/*v* exhibited the strongest conductive ability in MSCs through ALP and mineralization staining. In addition, they have demonstrated their ability in vivo and revealed a bone formation ability in the calvarial defect model. MeGC-MMT (MMT concentration: 1.5%) hydrogels were covered by new bone at almost 70%, which showed great potential for bone healing capability. Therefore, MMT-incorporated MeGC hydrogels provided good conditions for cell proliferation and differentiation, as well as the potential for bone tissue engineering.

Orive Group used hyaluronic acid and alginate to develop biologically active hydrogels and used montmorillonite (MMT) nanoclay to reinforce and stabilize the hydrogels [111]. The advantage of using MMT is enhancing osteogenic differentiation of human mesenchymal stem cells (hMSCs) even in a differentiation factor-free media. Moreover, MMT-incorporated hydrogels showed increased ALP enzyme activity and mineralization matrix formation, which indicates the hydrogel system is able to boost osteogenic differentiation and bone mineralization. In vivo studies demonstrated that the expression of OCN marker of late osteogenesis and mineralization in the newly formed bone showed the highest in the hydrogel embedded with SDF-1, compared to control groups. Therefore, the system provided a promising bone regeneration capacity for bone regeneration treatments.

Sumecton is a synthetic clay material with a chemical formula of MgAl_2_Si_4_O_10_(OH)_2_·nH_2_O. It belongs to the smectite group and is known for its high purity and transparency. The structure of Sumecton consists of a layered framework where magnesium and aluminum octahedral sheets are sandwiched between silicon tetrahedral layers, similar to other smectites [112,113]. This arrangement imparts a negative surface charge to the mineral due to the dominance of oxide anions. Sumecton is synthesized to be free from impurities, which enhances its performance in various applications [114]. Its high transparency makes it suitable for use in products where visual clarity is important. It is used in a range of fields, including cosmetics and paints, where it provides benefits such as high viscosity and clarity. Due to its properties, Sumecton is a valuable material in industrial applications and research, particularly for enhancing the performance of various products.

**Figure 5:**
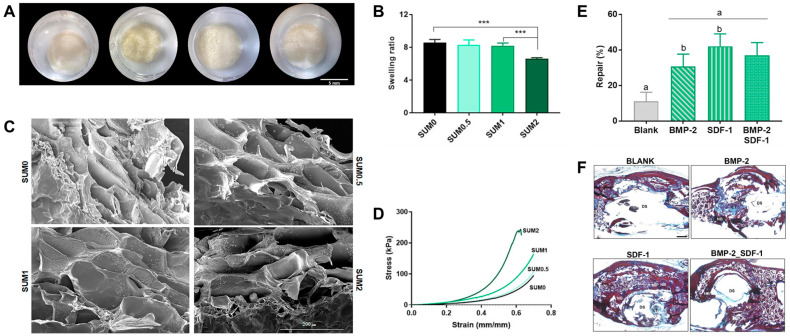
Gelatin–Sumecton composite hydrogels. (**A**) Macroscopical images of lyophilized hydrogels. Scale bar = 5 mm. (**B**) Swelling ratio after 24 h of hydration. Statistical significance: *** *p* < 0.001. (**C**) Scanning electron microscopy (SEM) representative images. Scale bar = 200 μm. (**D**) Compressive stress–strain curves of hydrogels. (**E**) Histomorphometric analysis showing the percentage of repair among all experimental groups. Same letters displayed in different histograms denote significant differences (*p* < 0.001) among these groups. (**F**) Cross-sectional representative images stained with VOF trichrome dye of the defect site. SUM0: gelatin hydrogel without Sumecton, SUM0.5: gelatin–Sumecton composite hydrogel with Sumecton 0.5%, SUM1: gelatin–Sumecton composite hydrogel with Sumecton 1%, SUM2: gelatin–Sumecton composite hydrogel with Sumecton 2%, CT: Connective tissue, DS: Defect site, NB: Newly formed bone. Scale bar is 500 μm in (**F**). Reproduced with permission from Lukin et al. [114].

Very recently, Lukin et al. developed a scaffold that consists of a gelatin-cross-linked network and synthetic nanoclay to enhance the inherent mechanical properties of a gelatin-based scaffold for bone regeneration [114]. In more detail, they used saponite as a type of nanoclay and designed saponite-derived synthetic nanoclay (Sumecton) which was incorporated into an enzymatically cross-linked gelatin network. Sumecton-reinforced gelatin-based hydrogels demonstrated that the swelling ratio was inversely proportional to the nanoclay concentration after 24 h of hydration, which indicates that higher nanoclay concentration decreased the swelling ratio of gelatin-based scaffolds (Figure 5A,B). In addition, the addition of nanoclay may increase cross-linking density and fiber arrangement that reduces pore size (Figure 5C). When the nanomaterial was incorporated into the hydrogels, there was an increased compressive strength (Figure 5D). In particular, nanoclay-composite hydrogels showed three times higher elastic modulus and ultimate stress compared to nanoclay-free hydrogels. Overall, the 3D systems offered osteoconductive properties in vitro and served as growth factor release platforms in vivo studies (Figure 5E,F).

**Table 2:** Sumecton and montmorillonite nanoclay-composite hydrogels for bone tissue engineering.

Type of Nanoclay	Type of Hydrogel	Bioactive Agent	Features	Ref.
Sumecton	Gelatin (bovine skin-derived, Type B)	SDF-1, bone morphogenetic protein-2 (BMP-2)	Enhanced mechanical properties of gelatin-based scaffolds by Sumecton nanoclay incorporation, osteoconductive properties in vitro, and ability of constructs to act as platforms for the release of growth factors in vivo	[114]
Montmorillonite	Thiol-modified hyaluronic acid, 8-arm PEGacrylate, alginate	Stromal cell-derived factor 1 (SDF-1)	Reinforce biocompatibility and osteogenic ability with nanoclay-composite, boost mineralization even in differentiation-free media, potential of hydrogels to mend bone and act as cell-carriers and delivery platforms for SDF-1, and in vivo enhanced capabilities of bone regeneration as well as of angiogenesis with SDF-1 delivery	[111]
Montmorillonite	Methacrylated glycol chitosan	None	Nanoclays can increase the Young’s modulus and slow down the degradation rate of hydrogels, promote proliferation, attachment, and differentiation of encapsulated mesenchymal stem cells, and enhance healing without additional therapeutic agents or stem cells in a critical-sized mouse calvarial defect model	[20]
Montmorillonite	Polycaprolactone, gelatin (bovine skin-derived, Type B), nanohydroxyapatite	None	Excellent mechanical properties but limited by hydrophobicity and long-term degradation, enhanced hydrophilicity, strength, adhesiveness, biocompatibility, biodegradability, and osteoconductivity by adding nanoclays, 3D printed structures with rectangular interconnected pores and well-distributed nanoclays, improved wettability, compressive strength, water uptake rate, biodegradability, and bioactivity, and enhanced cell proliferation, viability, and adherence	[115]

Halloysite nanotubes (HNTs) are inorganic nanoclay formed from rolled aluminosilicate kaolin sheets [116]. Their elongated shape and hydrophobic lumen make them suitable for entrapping hydrophobic drugs, acting as effective drug carriers [117]. Selective modification of HNTs’ inner (aluminum) and outer (silica) surfaces can improve their compatibility with polymer matrices and reduce the initial burst release of drugs in various recent studies (Table 3). In bone tissue engineering, HNTs are beneficial due to their ability to enhance mechanical properties of polymer matrices due to their high aspect ratio and robust structure [116]. Additionally, absorbable degradation products of HNTs can induce osteogenic cell differentiation.

The halloysite nanotube (HNT), a naturally occurring aluminosilicate nanotube, has been utilized by Huang et al. [118] to develop an HNTs-incorporated hydrogel for enhancing bone regeneration. The hydrogel was fabricated by photopolymerization using gelatin methacrylate and HNTs. The addition of HNTs in hydrogels can entrap a wide range of active agents within the inner lumen and void spaces in the aluminosilicate shell. Moreover, it could increase polymer strength and provide enhanced composite adhesion to the bone. Such HNTs incorporated hydrogel enhanced the adhesion and proliferation of human dental pulp stem cells and increased the osteogenic activity in bone formation both in vitro and in vivo. In addition, the group treated with HNTs/GelMA 5% and 7% hydrogels in calvarial defects showed the most bone formation and thickest mineralized bone formation than other groups, demonstrating a promising strategy for bone tissue engineering applications.

**Figure 6:**
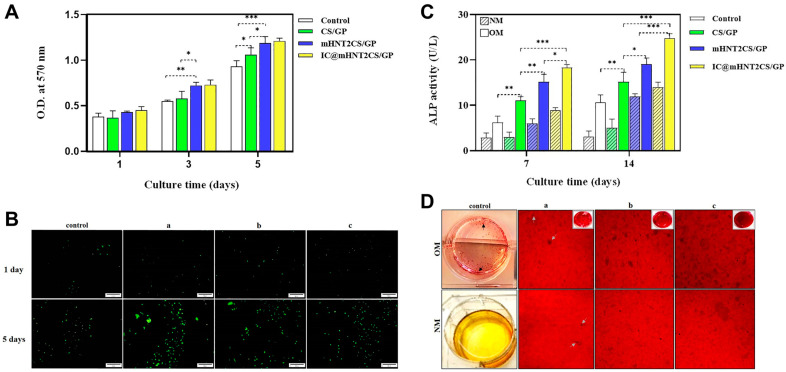
Halloysite nanotube-composite chitosan hydrogels. In vitro biocompatibility of scaffolds: (**A**) OD values; and (**B**) live/dead staining of free (control) and encapsulated hASCs in different hydrogels on days 1, 3, and 5 of culture. In vitro osteogenic differentiation of free (control) and encapsulated hASCs in normal medium (NM) and osteogenic medium (OM): (**C**) ALP activity on days 7 and 14 of culture; and (**D**) Alizarin red staining on day 21 of culture; scaffolds: (a) CS/GP = chitosan/β-glycerophosphate hydrogel, (b) mHNT2CS/GP = chitosan/β-glycerophosphate hydrogel with chitosan-modified HNTs, and (c) IC@mHNT2CS/GP = chitosan/β-glycerophosphate hydrogel with chitosan-modified HNTs and Icariin. * *p* < 0.03, ** *p* < 0.002, *** *p* < 0.001. Reproduced with permission from Aghdam et al. [119].

Aghdam et al. [119] developed chitosan-modified halloysite nanotubes (mHNTs) followed by icariin loading into mHNTs for a sustained drug release system. HNTs could provide a favorable effect on cellular adhesion, proliferation, and differentiation. Moreover, the surface of HNTs provided high compatibility with polymer matrices and reduced the initial burst release. It also boosted the mechanical properties of the polymer matrix and induced osteogenic cell differentiation. The mHNTs incorporated into chitosan hydrogel enhanced the mechanical strength and improved the proliferation of human adipose-derived stem cells (hASCs) encapsulated in the hydrogel (Figure 6A,B). Furthermore, the hydrogel system enhanced the differentiation of encapsulated hASCs into bone tissues followed by enhanced mineralization which could induce bone formation for bone regeneration (Figure 6C,D).

Ou et al. [120] fabricated a nanosilver/halloysite nanotubes/gelatin methacrylate (nAg/HNTs/GelMA) hybrid hydrogel to combine osteoimmunomodulatory properties and antibacterial activity for bone regeneration. The addition of nAg and HNTs enhanced mechanical performances with an increase in compressive modulus. In vitro studies confirmed that the hydrogel improved cell adhesion and spreading of human periodontal ligament stem cells (hPDLSCs). Under the inflammatory environment, hPDLSCs showed the greatest and almost three times higher osteogenic differentiation compared to the control. They demonstrated that the hydrogel showed a significantly enhanced osteoimmunomodulatory with antibacterial activities which prevented bacterial infection both in vitro and in vivo for treating infected bone defects for bone regeneration.

Alkaline phosphatase-halloysite (HAL-ALP) has been utilized in chitosan (CH) and chitosan–collagen (C-CH) hydrogel for bone regeneration [121]. Adding 10% of HAL-ALP in CH and C-CH could enhance cell viability which brings a positive effect of the formed mineral on osteoblasts. In addition, the presence of HAL nanotubes increased the porosity of CH and C-CH hydrogels with pore sizes suitable for cell adhesion, spread, interconnection, and proliferation for bone regeneration. Furthermore, the system can improve mechanical properties and promote biomineralization.

Zhou et al. [122] introduced clay halloysite nanotubes (HNTs) into sodium alginate (SA) to develop an SA/HNTs composite scaffold for bone regeneration. SA and HNTs were mechanically mixed to obtain a uniform bioink. 3D printing and high-temperature sintering were then applied to develop high-performance SA/HNTs ceramic scaffolds for bone repair. The addition of HNTs and sintering enhanced the scaffold’s surface roughness and improved cell adhesion. Moreover, the ceramic scaffold can regulate the osteogenic behavior of cells to enhance cell adhesion and spread. They demonstrated that such scaffolds could promote primary osteogenic differentiation, early mineralization, and late differentiation of bone marrow stem cells. Overall, the SA/HNTs hydrogel shows good biocompatibility and osteogenic activity as well as good bone repair abilities in vivo.

**Table 3:** Halloysite nanotubes (nanoclay)-composite hydrogels for bone tissue engineering.

Type of Nanoclay	Type of Hydrogel	Bioactive Agent	Features	Ref.
Halloysite nanotubes	Gelatin methacryloyl	None	Improve mechanical properties due to the incorporation of HNTs, maintain good cytocompatibility in vitro, upregulate expression of osteogenic genes and proteins in human dental pulp stem cells, and facilitate bone regeneration in rat calvarial defects	[118]
Halloysite nanotubes	Chitosan, glycerophosphate	Icariin	Increase mechanical strength by incorporating nanoclays into hydrogel, improve stem cell proliferation with nanoclay loading, enhance differentiation of stem cells into bone tissue, and sustain release of Icariin for a synergistic bone differentiation effect	[119]
Halloysite nanotubes	Gelatin methacryloyl	Nanosilver	Exhibit good biocompatibility with human periodontal ligament stem cells and macrophages, modulate inflammatory cytokines released by macrophages, enhance osteogenic differentiation of stem cells in an inflammatory environment, inhibit the growth of Gram-positive and Gram-negative bacteria, and better in vivo modulation of the osteoimmune microenvironment in the presence of nanosilver and effectively eliminate bacterial infection	[120]
Halloysite nanotubes	Chitosan, collagen type I (rat tail)	Alkaline phosphatase (ALP)	Significantly increase swelling in hydrogels with 30 wt% of nanoclay-ALP, increase scaffold porosity with composite of collagen and nanoclay-ALP, improve mechanical properties with nanoclays, reduce storage modulus with 20% collagen, and slow degradation in physiological pH	[121]
Halloysite nanotubes	Sodium alginate	None	Improve molding performance and good formability for 3D printing, good shape fidelity, printability, and mechanical properties after 3D printing, can be converted to a rigid ceramic scaffold at 1200 °C with good biocompatibility and osteogenic activity, and good rat calvarial bone repair abilities in vivo	[122]
Halloysite nanotubes	Polycaprolactone–polyethylene glycol-polycaprolactone, gelatin, nanohydroxyapatite (nHA), iron oxide nanoparticle (Fe_3_O_4_)	None	Increase mechanical performance by incorporating 3% HNT into hydrogels, enhance osteogenic activity with nanoclay, nHA, and Fe_3_O_4_	[123]

We conducted a Google patent search using the keywords “nanoclay”, “hydrogel”, and “bone”, resulting in the identification of 129 patents. These patents related to nanoclay-composite hydrogels encompass various aspects such as bioprinting devices for hydrogels in musculoskeletal therapy, regenerative bone healing scaffolds, and 3D printing techniques for bone tissue constructs. These patents emphasize the remarkable mechanical properties, biocompatibility, and osteogenic potential of nanoclay-composite hydrogels. Integrating these patented advancements could significantly enhance the development and clinical application of nanoclay-composite hydrogels in bone tissue engineering, effectively addressing challenges related to dispersion, scalability, and regulatory compliance.

## 5. Challenges and Future Perspectives

Despite the promising potential of nanoclay-composite hydrogels in bone tissue engineering, several challenges must be addressed to fully realize their clinical application. One of the primary obstacles is achieving optimal nanoclay dispersion within the hydrogel matrix. Poor dispersion can lead to agglomeration, which can adversely affect mechanical properties and uniformity of the composite. Advanced fabrication techniques such as high-energy mechanical mixing and surface modification of nanoclays may be required to ensure even distribution.

Scalability presents another significant challenge. While laboratory-scale synthesis and testing have shown positive results, scaling up the production process without compromising the material’s properties and performance remains a formidable task. Developing cost-effective and reproducible methods for large-scale production will be essential for translating these materials to clinical settings. Notably, the industrial-level reproducibility of nanoclay and nanoclay-composite hydrogels remains a critical issue. Achieving consistent quality and performance at a large scale requires rigorous control over raw materials, production processes, and environmental conditions. Variability in these factors can lead to inconsistencies in hydrogel properties, impacting their effectiveness and safety. Implementing robust quality control measures and standardized protocols for large-scale production is essential to ensure that nanoclay-based hydrogels meet the stringent requirements of industrial applications and can be reliably produced for clinical use.

Biocompatibility and long-term stability are crucial for the success of any biomaterial intended for implantation. Although in vitro and in vivo studies have demonstrated favorable cell adhesion, proliferation, and differentiation, long-term studies are needed to ensure that these materials do not elicit adverse immune responses or degrade in ways that could harm patients. Continuous evaluation and optimization of the hydrogel’s composition and structure will be necessary to meet these requirements.

Furthermore, understanding biological interactions at the molecular level is imperative for advancing nanoclay-composite hydrogels. Investigating how nanoclays influence cellular behaviors and signaling pathways will provide insights into designing more effective and responsive hydrogels. This knowledge will also help us customize hydrogels for specific applications, such as targeted drug delivery and regenerative therapies for different types of bone defects.

The integration of nanoclay-composite hydrogels with other emerging technologies, such as 3D bioprinting and bioactive molecule delivery systems, offers exciting future prospects. These combinations could lead to the development of more sophisticated and multifunctional scaffolds that can mimic the natural bone environment more closely.

Finally, a significant hurdle to the broad adoption of nanoclay-composite hydrogels is the complex process of securing approvals from regulatory authorities like the FDA and the European Medicines Agency (EMA). As an innovative medicinal cue, these hydrogels must undergo comprehensive assessments for safety, efficacy, and manufacturing consistency to comply with regulatory requirements. Additionally, developing standardized testing protocols and guidelines to evaluate the quality and performance of nanoclay-composite hydrogels is essential for achieving regulatory approval. The regulatory status of nanoclay-composite hydrogels varies across different markets. In the U.S., the FDA regulates these products under the medical devices category, requiring extensive safety and efficacy evaluations before approval. In the European Union, the EMA oversees similar regulatory processes, emphasizing compliance with the Medical Device Regulation (MDR). Additionally, in Asian markets like Japan and South Korea, the regulatory bodies ensure adherence to local safety and quality standards, with specific guidelines for nanomaterials. Overall, achieving regulatory approval involves rigorous testing and documentation to ensure the safety and effectiveness of nanoclay-composite hydrogels in clinical applications. Detailed considerations are as follows. The design of hydrogels must ensure appropriate materials and physico-chemical characteristics. They should adhere to satisfactory microbiological standards and maintain uniform content. High-quality manufacturing, safety, and packaging are essential, as are safe, nonhazardous procedures for their use. Material safety is crucial for long-term use. Clear labeling with usage instructions is necessary. Hydrogels should be applied with a suitable applicator for proper delivery, ensuring consistent results without complications. Immunological safety must also be considered.

Collaborative efforts between researchers, clinicians, and regulatory bodies will be crucial to navigating complex pathways of approval and ensuring that these innovative materials can be safely and effectively used in patient care. Future research should focus on overcoming these hurdles, paving the way for clinical applications that can significantly enhance bone regeneration strategies. Additionally, looking ahead, future directions in bone tissue engineering are poised to capitalize on emerging technologies such as stem cell engineering, gene editing, and advanced biomaterials design to overcome current challenges and revolutionize clinical practice. Harnessing the power of regenerative medicine, tissue engineering, and biotechnology, researchers are trying to develop next-generation therapies capable of restoring function to damaged or diseased bone tissue with unprecedented precision and efficacy.

## 6. Conclusions

Regenerative engineering is an interdisciplinary field that integrates principles of tissue engineering, materials science, and regenerative medicine to restore or replace damaged tissues and organs. This approach hinges on three major components: scaffold, cells, and bioactive signals. Among innovative materials explored for scaffolding, nanoclay has garnered significant attention due to its unique properties and versatility. Scaffolds provide structural support for tissue formation. Nanoclay has been utilized to create various types of scaffolds such as hydrogels and porous structures. These nanoclay-composite hydrogels are notable for their mechanical strength, biocompatibility, and ability to mimic the extracellular matrix, thereby positively controlling cell attachment, growth, and fate. Nanoclay can directly interact with cells, exhibiting bioactive properties that can modulate cell behavior. These interactions can be tailored to control various cell functions, such as proliferation, differentiation, and migration, which are crucial for effective tissue regeneration. Additionally, nanoclay’s charged and structural characteristics enable it to sequester and release a variety of bioactive agents, including small molecule drugs and large proteins. This controlled release of therapeutic agents can be crucial for promoting tissue regeneration and healing. Emerging applications of nanoclay in regenerative medicine include bone regeneration and advanced drug delivery systems. Nanoclay offers a multifaceted platform for regenerative engineering, with its application in scaffold, cell modulation, and bioactive signal delivery, showcasing its potential to revolutionize bone tissue engineering and regenerative medicine. By harnessing these properties, researchers and clinicians can develop more effective and targeted therapies for a wide range of medical conditions.

## Data Availability

Not applicable.
